# Exploring associations between gaze patterns and putative human mirror neuron system activity

**DOI:** 10.3389/fnhum.2015.00396

**Published:** 2015-07-14

**Authors:** Peter H. Donaldson, Caroline Gurvich, Joanne Fielding, Peter G. Enticott

**Affiliations:** ^1^School of Psychological Sciences, Monash University, ClaytonVIC, Australia; ^2^Monash Alfred Psychiatry Research Centre, The Alfred and Central Clinical School, Monash University, MelbourneVIC, Australia; ^3^Cognitive Neuroscience Unit, School of Psychology, Deakin University, BurwoodVIC, Australia

**Keywords:** transcranial magnetic stimulation, mirror neurons, motor resonance, autism, gaze pattern, predictive gaze

## Abstract

The human mirror neuron system (MNS) is hypothesized to be crucial to social cognition. Given that key MNS-input regions such as the superior temporal sulcus are involved in biological motion processing, and mirror neuron activity in monkeys has been shown to vary with visual attention, aberrant MNS function may be partly attributable to atypical visual input. To examine the relationship between gaze pattern and interpersonal motor resonance (IMR; an index of putative MNS activity), healthy right-handed participants aged 18–40 (*n* = 26) viewed videos of transitive grasping actions or static hands, whilst the left primary motor cortex received transcranial magnetic stimulation. Motor-evoked potentials recorded in contralateral hand muscles were used to determine IMR. Participants also underwent eyetracking analysis to assess gaze patterns whilst viewing the same videos. No relationship was observed between predictive gaze and IMR. However, IMR was positively associated with fixation counts in areas of biological motion in the videos, and negatively associated with object areas. These findings are discussed with reference to visual influences on the MNS, and the possibility that MNS atypicalities might be influenced by visual processes such as aberrant gaze pattern.

## Introduction

Discovered serendipitously whilst recording single motor neurons in macaques, mirror neurons are cells that fire both when an action is performed, and when that same action is observed (Di Pellegrino et al., 1992). Homologous neurons and mechanisms have been inferred in humans via a range of techniques, with the inferior parietal lobule (IPL) and the inferior frontal gyrus (IFG) typically implicated. These structures, along with the superior temporal sulcus (STS), are together referred to as the parietofrontal mirror neuron system (MNS; Iacoboni and Mazziotta, 2007). Some assert that the MNS is responsible for, or implicated in, social cognitive processes such as action/goal/intention understanding (Gallese et al., 2013), imitation (Williams et al., 2001), empathy (Iacoboni and Mazziotta, 2007), and theory of mind (Perkins et al., 2010). Others have challenged these claims (Dinstein et al., 2008; Hickok, 2009).

These potential links between the MNS and social cognition have led to hypotheses such as the broken mirror theory of autism spectrum disorder (ASD). This posits that if MNS function is impaired, this might lead to a decreased ability to understand and/or imitate what we observe, which would in turn contribute to the social and mentalising deficits characteristic of ASD (Ramachandran and Oberman, 2006). Relevant results in this regard are inconsistent, however. One possible explanation for such inconsistency is that MNS dysfunction may be secondary, influenced by factors such as atypical visual processing. The MNS necessarily receives input from occipital and temporal regions involved in visual processing. It has been suggested that abnormalities of the STS (a region inputting to the MNS and involved in integrating visual biological motion cues), for example, may provide a neural basis for aberrant social cognitive performance (Dakin and Frith, 2005; Hickok, 2013). More generally, MNS activity (both in the immediate and longer term) in the broader population is likely to be influenced by the quality of visual input, which is in turn shaped by factors such as gaze variables and early visual processing.

Two relevant gaze variables in this regard are gaze pattern (the number and duration of fixations in particular areas of a visual scene) and predictive gaze (PG; eye movements that proactively arrive at a visual target before a moving stimuli does). Given that mirror neurons are most active in response to observing transitive actions (Rizzolatti and Craighero, 2004), it could be hypothesized that gaze patterns that attend relatively strongly to the biological motion aspect of a transitive action (rather than the object being acted upon) would be more likely to promote greater MNS activity. Regarding PG, Flanagan and Johansson (2003) found similar PG profiles when participants observed and when they performed the same action. They inferred that observers implement eye motor programs directed by their own motor representations of the performance of that action. This inference has been supported by others and hypothesized to be related to MNS activity (Falck-Ytter et al., 2006; Elsner et al., 2013).

Whilst this proposed influence of such gaze variables on MNS activity may seem straightforward, the relationship has not been empirically examined in humans as far as the authors are aware. One study did explore this relationship in monkeys, however. Maranesi et al. (2013) recorded premotor cortex mirror neuron activity (via microelectrodes) and eye position in macaque monkeys during the observation of grasping actions. Most neurons recorded discharged more strongly when the monkey was looking directly at the action, and discharged earlier and more strongly when the gaze was proactive rather than reactive. The authors therefore concluded that mirror neuron activity was related to gaze behavior.

Direct electrode recordings of mirror neurons are generally not possible in humans. One paradigm used to putatively measure MNS activity employs transcranial magnetic stimulation (TMS) during the observation of transitive actions. TMS is a non-invasive means of stimulating the cortex via pulsed magnetic fields. When applied to the ‘hand region’ of the primary motor cortex, TMS can be used to elicit a motor evoked potential (MEP) in contralateral hand muscle which can be measured by electromyography (EMG). The observation of another’s hand movement during such TMS application results in a facilitated (i.e., increased) MEP (Fadiga et al., 1995; Theoret et al., 2005). In this context, MEPs are regarded as a measure of interpersonal motor resonance (IMR), a concept alluding to the activity of one’s motor/sensorimotor system whilst viewing another’s motor actions (Uithol et al., 2011). IMR is in turn an index of putative MNS activity.

The current study utilized this TMS paradigm to investigate the human mirror neuron response, whilst eye tracking recordings were also taken to investigate aspects of gaze and visual processing during action observation. PG times [*(time of hand or object arrival at target) – (time of first fixation on target after the first fixation on arriving hand or object)*] and fixations in areas of interests (AOIs) were measured, with the latter focusing on hand movement areas versus object areas. It was hypothesized that IMR would be positively associated with PG times and fixation counts in hand AOIs, and negatively associated with fixation counts in object AOIs.

## Materials and Methods

### Participants

**FIGURE 1:**
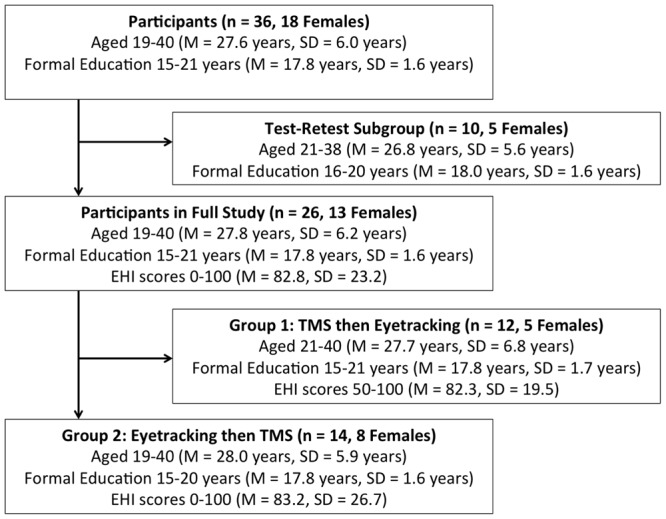
**Participant Demographics and Subgroup Assignment.** EHI, Edinburgh Handedness Inventory. TMS, transcranial magnetic stimulation.

As per approval conditions of the Alfred Ethics Committee and the Monash University Human Research Ethics Committee, participants were recruited via advertisements distributed throughout the Alfred Hospital and Monash University managed sites. Participants were 36 healthy adults aged 18–40 years (*M* = 27.6, SD = 6.0, 18 females; **Figure 1**).

Written informed consent was obtained, and participants were reimbursed $20 for the full study (both TMS and eyetracking) or $10 for the test–retest component (eyetracking only). Participants were without hard contact lenses, and reported having no neurological, psychiatric, or ocular condition or syndrome. Participants were also screened to ensure they met TMS safety criteria (Rossi et al., 2009).

### Materials

The Edinburgh Handedness Inventory (EHI) is a 10-item index of a person’s left or right hand dominance in everyday activities, such as writing or using cutlery (Oldfield, 1971). It is the most widely used such measure (Fazio et al., 2011), and possesses strong psychometric properties (McFarland and Anderson, 1980). Scores range from -100 to +100, with scores of +40 or above categorizing the person as right handed.

### Procedure

#### Full Study (TMS and Eyetracking)

Participants in the full study completed the EHI, and forms related to TMS screening criteria and demographic information. TMS and eyetracking components were conducted separately, in a counterbalanced manner (see **Figure 1**).

##### TMS component

As done previously (Enticott et al., 2012b), IMR was measured by delivering single TMS pulses to the primary motor cortex of the left hemisphere and recording, via EMG, the MEP of the contralateral first dorsal interosseous (FDI) and abductor digiti minimi (ADM) muscles during the observation of short video clips. Videos displayed either a static right hand (baseline condition), or right handed object-grasping actions. Such transitive actions are understood to promote mirror neuron activity in healthy participants (Rizzolatti and Craighero, 2004). As discussed in the introduction, MEPs in this context are regarded as a measure of IMR, a concept alluding to the activity of one’s motor/sensorimotor system whilst viewing another’s motor actions (Uithol et al., 2011). IMR is in turn an index of putative mirror neuron activity in the premotor cortex.

Transcranial magnetic stimulation was administered using a Magstim-200 stimulator (Magstim Company Ltd., UK), with a 70 mm figure-of-eight coil placed against the scalp of the left primary motor cortex in the usual manner. The site of maximum FDI stimulation was found by observing the greatest post-pulse EMG response in the FDI. Resting motor thresholds (RMTs) were defined as the lowest TMS intensity that elicited an EMG response in the FDI of approximately 1 mV over five consecutive trials.

Electromyography was recorded from adhesive surface electrodes placed over the participant’s right FDI and ADM muscles. EMG signals were filtered (high pass of 10 Hz, low pass of 500 Hz) and amplified using Powerlab/4SP (AD Instruments, Colorado Springs, CO, USA). Sampling was done using a CED Micro 1401 mk II analog-to-digital converting unit (Cambridge Electronic Design, Cambridge, UK).

**FIGURE 2:**
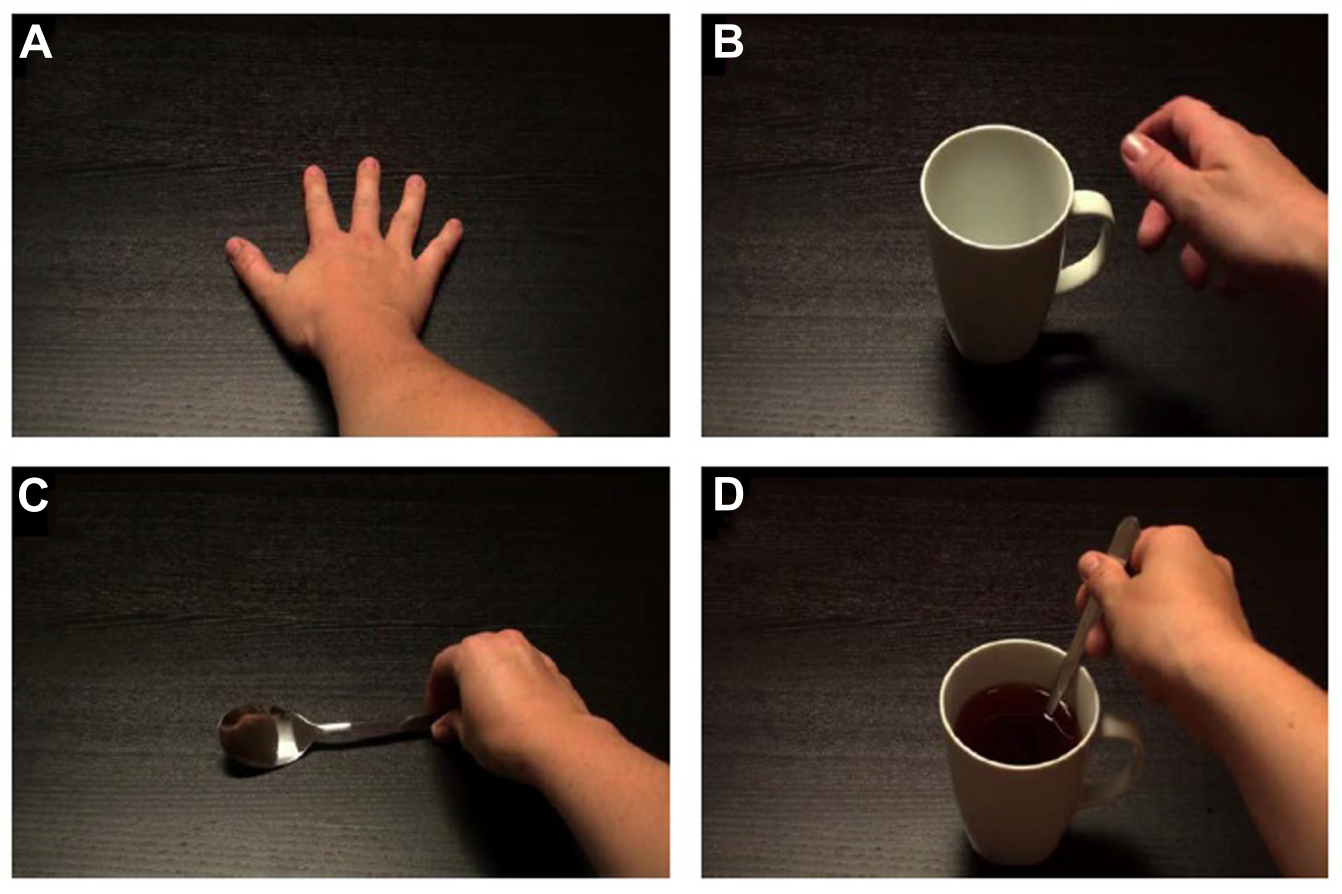
**Screenshots of static/grasping hand videos. (A)** Static Hand; **(B)** Mug Grasp; **(C)** Spoon Grasp; **(D)** Tea Stir.

Participants viewed the stimuli on a 22-inch LCD monitor (aspect ratio of 16:9), positioned 120 cm ahead of the individual, at eye-level. Stimuli were two quasi-random blocks of 60 short videos (4 s each), of 6 min and 10 s duration per block. Participants had a break of 1–5 min between blocks. Both blocks had a 12 s blank-screen lead-in, and blank-screen intervals of 2 s between videos. There were four video types of which there were ‘late’ and ‘early’ versions of each (**Figure 2**). These will be referred to as Static Hand, Mug Grasp, Spoon Grasp, and Tea Stir. Videos produced small flashes of light in the top left corner of the image at specific times. For ‘late versions’, the flash immediately preceded grasp-completion. For the ‘early versions’, the flash occurred earlier. A photodiode (embedded in a flash-sized occlusion pad) detected the flash in each video, triggering a single TMS pulse at 100% of RMT. A second experimenter monitored participant attendance to the stimuli and appropriateness of EMG output.

##### Eyetracking component

**FIGURE 3:**
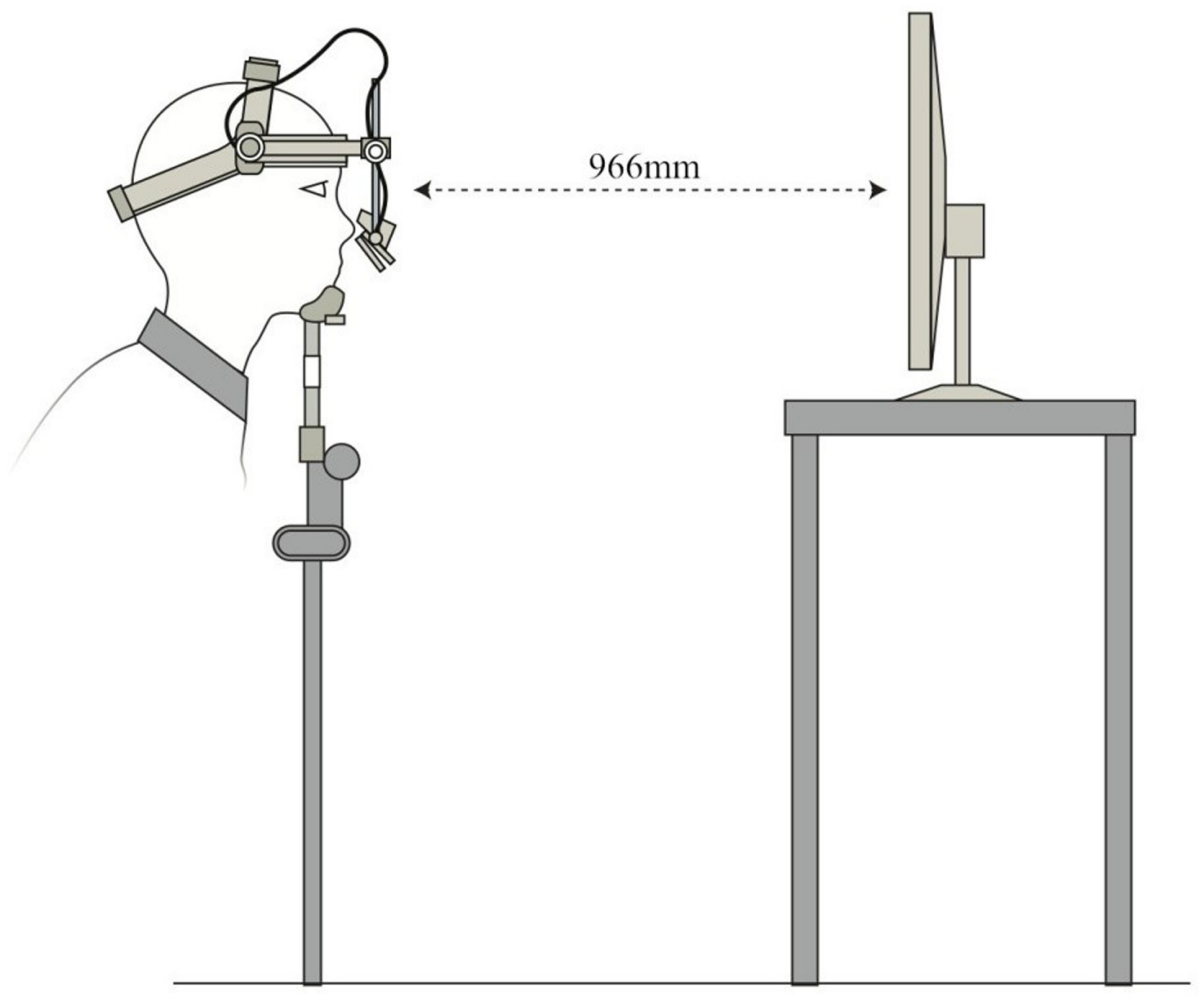
**Schematic diagram of Eyelink II setup for eyetracking tasks**.

Eyetracking tasks were created using Experiment Builder software (SR Research, Ontario). Eye movements were tracked using an Eyelink II head-mounted eye-tracker (SR Research, Ontario), which recorded gaze at 500 Hz with an average accuracy of 0.5°. Stimuli were presented on a 1024 × 768 resolution, 303 mm × 378 mm LED screen positioned 966 mm in front of the participants at eye-level. A chinrest minimized head movement (**Figure 3**). Glass-wearing participants wore glasses in both TMS and eyetracking sessions. Camera setup and calibrations were done in moderate light, and the tasks themselves were completed in dark conditions. Drift corrections were performed at regular intervals throughout the two tasks.

First, after a five-point-calibration, participants performed a basic prosaccade task, to ensure there were no gross ocular motor abnormalities in the capacity to track visual stimuli. A green target cross, subtending 1° of visual angle, was presented at the center of the screen for either 1000 or 1500 ms. This was followed by the appearance of the target cross at one of four peripheral locations (±5 or 10°) from the center of the screen, remaining for either 1250, 1500, or 1750 ms. Participants were instructed to look at the center of the target cross each as soon as it appeared on the screen. Targets appeared randomly in four blocks of 12 trials each, with rest breaks provided between each block.

Second, after a nine-point-calibration, an occlusion pad was placed over the flash location. Participants viewed the same grasping/static hand videos as used in the TMS session; 24 randomized repeats of each of the four videos were presented. There was a pause every 16 videos, to rest and re-calibrate where necessary. Task duration was circa 5 min and 30 s, designed to approximate the length of blocks in the TMS component. No instructions were given (in this or the TMS component) regarding how to view the videos. If participants asked for instructions they were told to view the videos as they wished.

#### Test–Retest Group (Eyetracking Only)

As TMS and eyetracking components were conducted separately in the full study, the immediate temporal relationship between eyetracking variables and IMR was not measured. To determine whether gaze patterns were stable over time, a test–retest eyetracking paradigm was also conducted. This involved a separate group (*n* = 10) who did not undergo the TMS component, and had not witnessed the stimuli previously. They performed the first (prosaccade) and second (grasping/static hand videos) eyetracking tasks as outlined above. They then rested for a period emulating the gap between the eyetracking and TMS components of the full study (approximately 10 min), before repeating the second task.

### Data Analysis

#### Full Study Group (TMS and eyetracking)

For the TMS component, trials exhibiting tonic muscle activity within 200 ms before TMS pulse administration were removed prior to analysis (3.2% of all trials). Median MEP readings for static and transitive hand conditions were utilized to calculate Motor evoked potential percentage change (MEP-PC) values, as done previously (Enticott et al., 2012a). This provides a relative IMR index, with larger MEP-PCs indicative of greater responses in active compared to static hand conditions. MEP-PC was calculated as: *MEP-PC = [(MEP transitive – MEP static)/MEP static]^∗^100.* One sample Wilcoxin signed-ranks tests compared MEP-PC for the different images and muscles with zero, to determine where significant facilitation had occurred.

**FIGURE 4:**
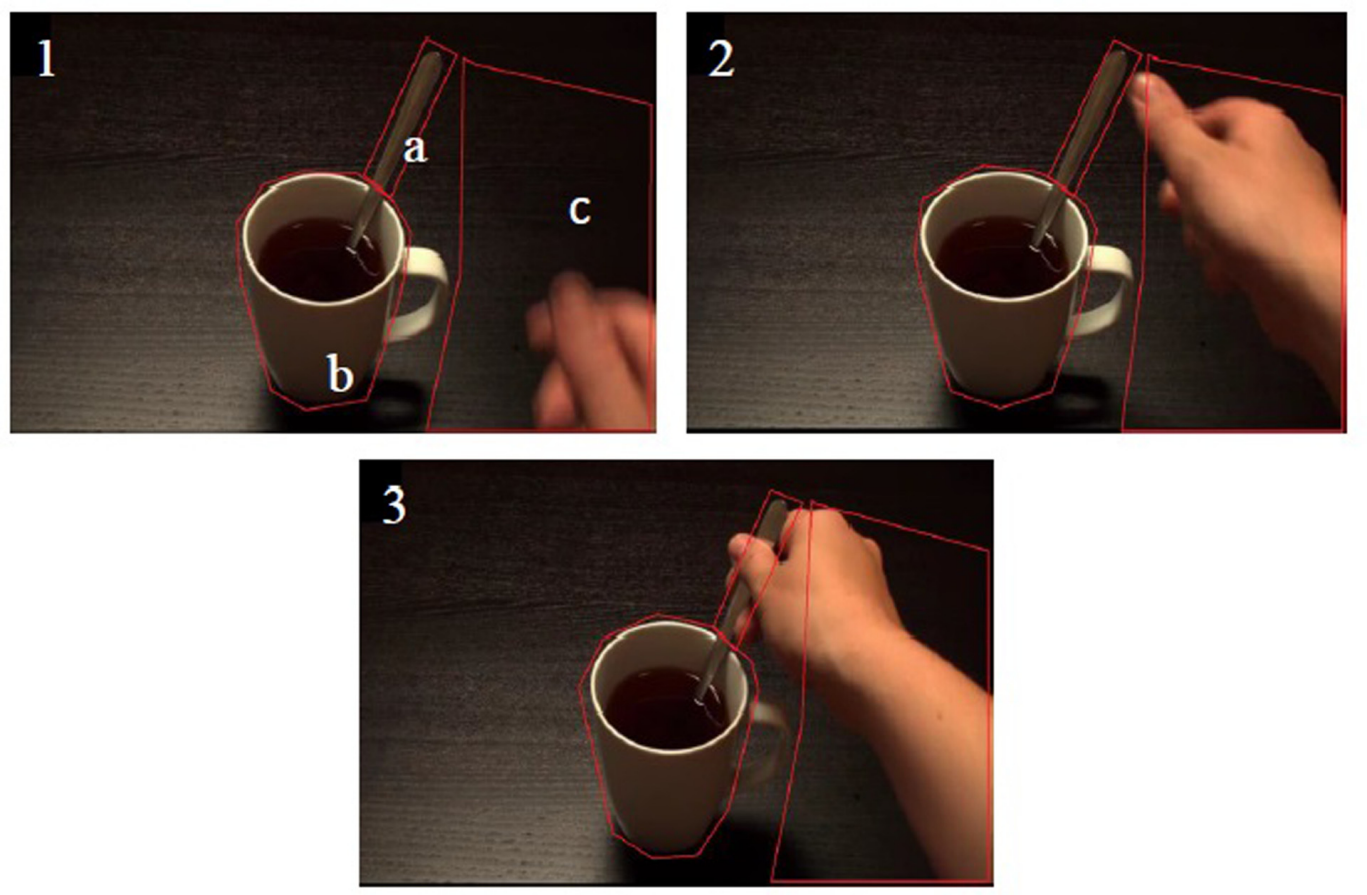
**Areas of interest (AOI) at three consecutive time points for the Tea Stir video.** (a) Spoon Handle; (b) Mug Body; (c) Total Hand.

For the eyetracking component, PG was calculated as: *PG (in milliseconds) = (time of hand or object arrival at target) – (time of first fixation on target after the first fixation on arriving hand or object)*. Larger PG values indicate earlier PGs. Eyelink systems define a fixation as anything that is not a saccade or a blink. Fixation reports for the AOIs (see **Figure 4** for the Tea Stir AOIs as an example, which displays the emphasis on hand-related versus object-related areas) were obtained, providing the number of fixations in each AOI for each trial. These were then averaged across all trials for each participant. Spearman’s correlations were used to examine relationships between Mean MEP and eyetracking variables (PG times and fixations in AOIs). An alpha level of 0.01 was chosen. This does not constitute a full Bonferroni adjustment, but was considered a reasonable level of reduction of family wise type 1 error risk that would still enable the possibility of detecting relationships in these exploratory correlations.

Independent-samples Mann–Whitney *U* tests were used to assess differences between Group 1 (TMS first) and Group 2 (eyetracking then TMS), to determine whether order effects were present. Subsequent Spearman’s correlations between Mean MEP and eyetracking variables were then re-calculated on an individual group basis (α = 0.01, adjusted for multiple comparisons).

To assess whether Mean MEP and eyetracking scores were influenced by participants’ underlying sensorimotor function, Spearman’s correlations were used to examine the relationship between prosaccade variables (accuracy and latency) and Mean MEP and eyetracking variables (α = 0.01, adjusted for multiple comparisons).

As a final exploratory analysis, Spearman’s correlations were calculated to relate Mean MEP and eyetracking variables to *fixation percentages* in each AOI (α = 0.01, adjusted for multiple comparisons). Fixation percent refers to the percentage of overall fixations that fall in a given AOI over the duration of a given trial. This was done to provide a comparative index of relative interest in each AOI, as *fixation numbers* in AOIs may relate to general attentional factors or activity in terms of rate of saccadic movements, rather than (or as well as) interest in particular AOIs.

#### Test–Retest Group (Eyetracking then Eyetracking)

Spearman’s correlations (α = 0.01) were calculated to compare AOI fixation counts in the first and second viewing sessions of the static/grasping hand videos.

## Results

Descriptive statistics are summarized in **Figure 1**. All statistical analyses were performed in SPSS (Version 20.0).

### Mean MEP

As shown in **Table 1**, MEP-PC, (our measure of putative mirror system activity) for the stimulus “Spoon Grasp Early” yielded the most consistent facilitation profile for both FDI and ADM, and were therefore averaged to produce a single MEP-PC value for subsequent correlational analyses (this will be referred to as Mean MEP). Mean MEP distribution was positively skewed, and this was not significantly rectified following log transformation.

**Table 1:** One-sample Wilcoxin signed rank test results comparing MEP-PC values to 0.

Video	Median MEP-PC^c^	Significance (*p*)
*FDI^a^ Spoon Grasp Early*	*12.31^d^*	*0.058*
FDI Spoon Grasp Late	-2.62	0.568
FDI Tea Stir Early	-1.58	0.439
FDI Tea Stir Late	8.57	0.107
FDI Mug Grasp Early	-4.99	0.551
FDI Mug Grasp Late	-7.37	0.751
*ADM^b^ Spoon Grasp Early*	*12.51^d^*	*0.005^∗∗^*
ADM Spoon Grasp Late	-9.10	0.009^∗∗^
ADM Tea Stir Early	5.95	0.073
ADM Tea Stir Late	-1.00	0.751
ADM Mug Grasp Early	1.86	0.341
ADM Mug Grasp Late	-11.34	0.026^∗^

*^a^FDI, first dorsal interosseus muscle.*

*^b^ADM, abductor digitiminimi muscle.*

*^c^MEP-PC, motor evoked potential percent change.*

*^d^Italicized content refers to items contributing to mean MEP calculation.*

*^∗^*p* < 0.05, ^∗∗^*p* < 0.01.*

### Predictive Gaze (PG) and Mean MEP

Predictive gaze was calculated as: *PG (in milliseconds) = (time of hand or object arrival at target) – (time of first fixation on target after the first fixation on arriving hand or object)*. Larger PG values indicate earlier PGs. No significant relationships were found between Mean MEP and average PG times in any of the static/grasping videos (**Table 2**). There was a trend toward a negative relationship in the Tea Stir (*r* = -0.77, *p* = 0.072) video, though only six participants displayed PG during viewing. In the Spoon Grasp video, no PGs were recorded.

**Table 2:** Spearman’s correlation coefficients for average predictive gaze (PG) times and mean MEP.

	Mug Grasp	Tea Stir	Ball Roll	Mug Pass	Long Mug Grasp	Pen Grasp
Mean MEP	-0.31	-0.77	0.25	0.18	-0.40^∗^	-0.15

*^∗^*p* < 0.05.*

### Areas of Interest (AOIs) and Mean MEP

**FIGURE 5:**
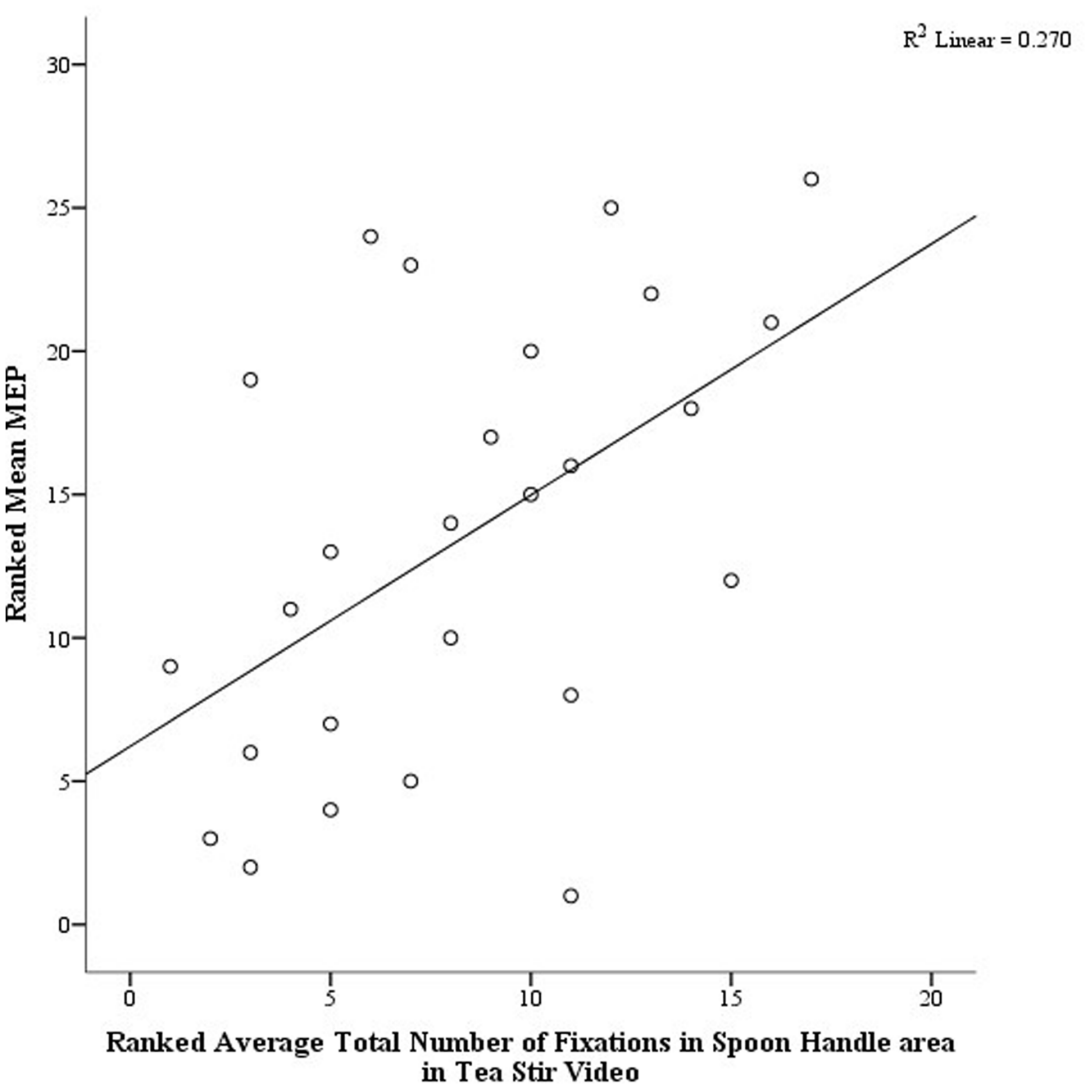
**Scatterplot displaying the linear relationship between ranked Mean MEP values and ranked Fixation Counts in the Spoon Handle area of interest in the Tea Stir video**.

**FIGURE 6:**
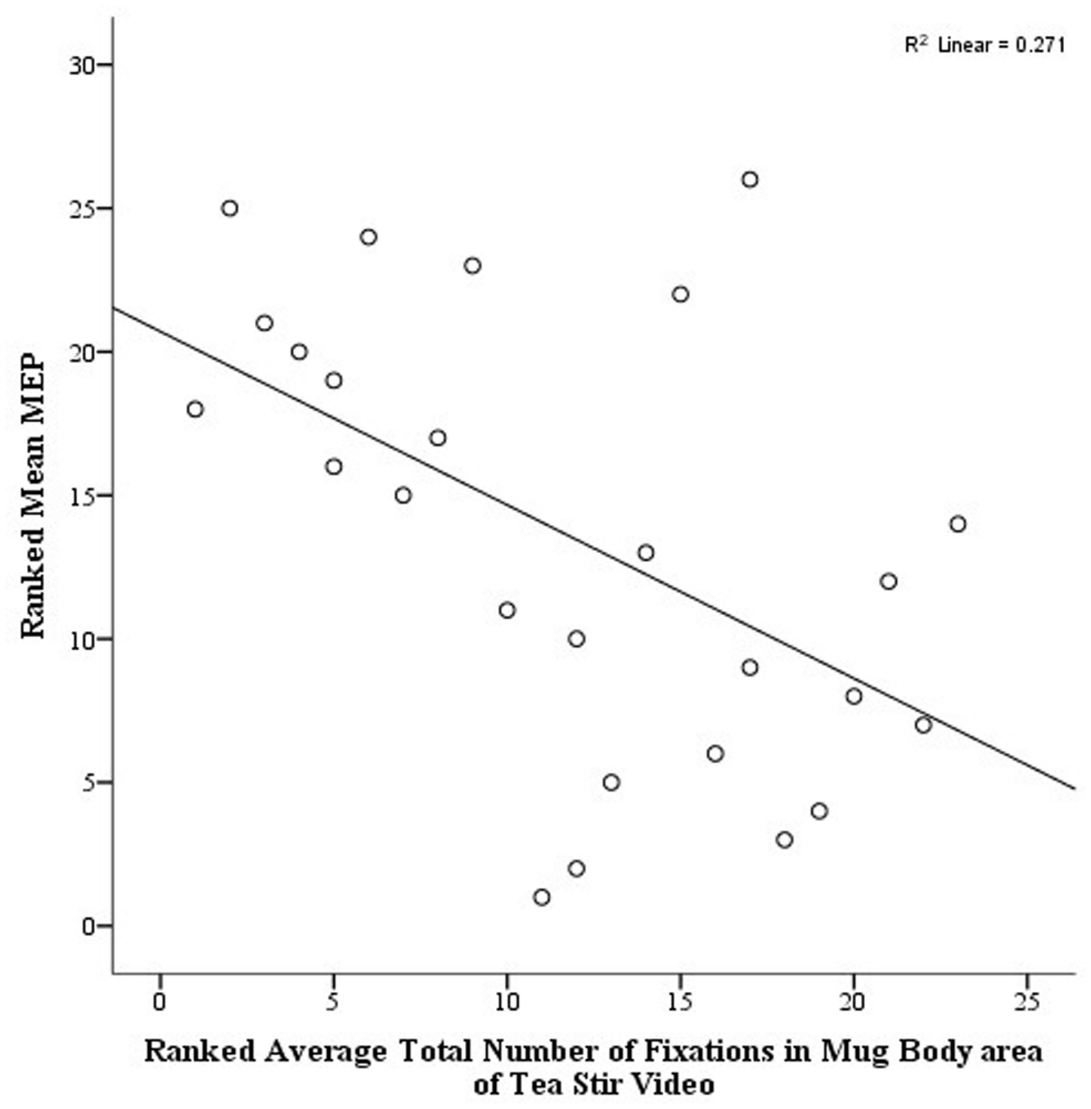
**Scatterplot displaying the linear relationship between ranked Mean MEP values and ranked Fixation Counts in the Mug Body area of interest in the Tea Stir video**.

As noted in the methodology, Eyelink systems define a fixation as anything that is not a saccade or a blink. Fixation reports for the AOIs (see **Figure 4** for the Tea Stir AOIs as an example, which displays the emphasis on hand-related versus object-related areas) were obtained. These reveal the number of fixations in each AOI for each trial, which were then averaged across all trials for each participant. Two relationships were significant at α = 0.01 (**Table 3**). Mean MEP was found to have a negative relationship with fixation number in the Mug Body AOI (*r*_s_ = -0.52, *p* = 0.006, two-tailed, *n* = 26), and a positive relationship with fixation number in the Spoon Handle AOI (*r*_s_ = 0.51, *p* = 0.007, two-tailed, *n* = 26). These were large effects, with fixation numbers in these cases accounting for approximately 27 and 26% of variance in Mean MEP, respectively (**Figures 5** and **6**). There were also trends toward a positive relationship between Mean MEP and Total Hand AOI fixation number (*p* = 0.049), and negative relationships between Mean MEP and Total Mug and Total Spoon AOIs (*p* = 0.034 and *p* = 0.082, respectively). Overall, hand AOI fixations were more likely to be positively associated with Mean MEP, and object AOI fixations negatively associated. It should also be noted, however, that non-linear relationships not tested for here (and elsewhere in this study) may also exist.

**Table 3:** Spearman’s correlation coefficients for area of interest fixation counts and mean MEP.

	Mug Grasp		Spoon Grasp		Tea Stir		
	Total Hand	Total Mug	Total Hand	Total Spoon	Total Hand	Mug Body	Spoon Handle
Mean MEP	0.17	-0.42^∗^	-0.18	-0.35	0.39^∗^	-0.52^∗∗^	0.51^∗∗^

*^∗^*p* < 0.05, ^∗∗^*p* < 0.01.*

### Order Effects

A significant difference was found in Mean MEP scores between Group 1 who did TMS first (*Md* = 4.3, *n* = 12) and Group 2 who did eyetracking first (*Md* = 22.9, *n* = 14), *U* = 33, *z* = -2.62, *p* = 0.008, two-tailed, *r* = 0.51, a large effect. Subsequent individual group-by-group Mean MEP and AOI correlations are summarized in **Table 4**. Relationships were all non-significant at the subgroup level (adjusted alpha of 0.01). However, there remained a trend toward a negative relationship between Mean MEP and Mug Body fixation count in Group 1 for the Tea Stir video (*p* = 0.026). No significant PG time differences were observed between Group 1 and Group 2 at α = 0.01.

**Table 4:** Spearman’s correlation coefficients for area of interest fixation counts and mean MEP in Groups 1 and 2.

	Mug Grasp		Spoon Grasp		Tea Stir		
	Total Hand	Total Mug	Total Hand	Total Spoon	Total Hand	Mug Body	Spoon Handle
Mean MEP (Group 1, TMS first)	0.01	-0.46	-0.07	-0.37	0.17	-0.64^∗^	0.33
Mean MEP (Group 2, TMS second)	0.28	-0.24	-0.09	-0.10	0.34	-0.35	0.31

*^∗^*p* < 0.05.*

### Prosaccade Task Relationships

There were no significant relationships between prosaccade variables and Mean MEP or AOI fixation counts at α = 0.01 (**Table 5**), suggesting scores were not unduly impacted by participants’ underlying sensorimotor function.

**Table 5:** Spearman’s correlation coefficients for area of interest fixation counts, mean MEP, and prosaccade variables.

	Mug Grasp		Spoon Grasp		Tea Stir			Mean MEP
	Total Hand	Total Mug	Total Hand	Total Spoon	Total Hand	Mug Body	Spoon Handle	
Latency	0.08	-0.19	0.11	-0.20	0.05	-0.29	0.29	0.29
Accuracy (primary saccade)	0.45^∗^	0.13	0.11	-0.07	0.24	0.16	0.32	0.01
Accuracy (final eye position)	0.02	-0.04	0.38	0.13	0.06	-0.20	-0.28	-0.03

*^∗^*p* < 0.05.*

### Fixation Percentages in AOIs

Fixation percentage (the averaged percent of fixations that fell in each AOI during the duration of each trial) was found to have a positive relationship with Mean MEP in the Spoon Handle AOI (*r*_s_ = 0.52, *p* = 0.006, two-tailed, *n* = 26). This was a large effect, accounting for approximately 27% of variance in Mean MEP. All other relationships were non-significant at α = 0.01 (**Table 6**), though there were two other trends in the Tea Stir video AOIs. Mean MEP approached a positive relationship with Total Hand fixation percent (*p* = 0.070), and a negative relationship with Mug Body fixation percent (*p* = 0.082).

**Table 6:** Spearman’s correlation coefficients for area of interest fixation percentages and mean MEP.

	Mug Grasp		Spoon Grasp		Tea Stir		
	Total Hand	Total Mug	Total Hand	Total Spoon	Total Hand	Mug Body	Spoon Handle
Mean MEP	0.15	-0.11	-0.18	-0.25	0.36	-0.35	0.52^∗∗^

*^∗∗^*p* < 0.01.*

### Test-Retest Group (Eyetracking then Eyetracking)

Spearman’s correlations suggested that object fixation numbers were more temporally stable than hand fixation rates (**Table 7**). Two object areas reached significance at α = 0.01, and two spoon areas in the relevant videos also approached significance (*p* = 0.029 for Total Spoon, and *p* = 0.024 for Spoon Handle).

**Table 7:** Spearman’s correlation coefficients for fixation counts in areas of interest (AOI) in test–retest group.

		Cup Grasp Retest		Spoon Grasp Retest		Tea Stir Retest		
		Total Cup	Total Hand	Total Hand	Total Spoon	Total Hand	Mug Body	Spoon Handle
Cup Grasp	Total Cup	0.88^∗∗^						
	Total Hand		0.36					
Spoon Grasp	Total Hand			-0.17				
	Total Spoon				0.69^∗^			
Tea Stir	Total Hand					0.35		
	Mug Body						0.77^∗∗^	
	Spoon							0.70^∗^

*^∗^*p* < 0.05, ^∗∗^*p* < 0.01.*

## Discussion

In the present study TMS and eyetracking technologies were used to examine the relationship between IMR (motor cortical facilitation while observing motor actions) and gaze pattern during the observation of grasping actions. No relationship was found between PG and Mean MEP. However, fixation counts in several AOIs were found to have strong associations with Mean MEP, our index of IMR. It should also be noted, however, that only linear relationships were examined here. Non-linear relationships not tested for may also exist.

The non-significant PG result found here is not necessarily inconsistent with prior research. Although the rationale for a relationship between PG and MNS activity is well established (Flanagan and Johansson, 2003; Falck-Ytter et al., 2006; Elsner et al., 2013), no previous studies have directly linked PG to a neurophysiological measure of IMR in humans. Furthermore, PG results in clinical samples with theorized MNS deficits (such as ASD) have been mixed (Falck-Ytter, 2010; Falck-Ytter et al., 2012). One study found a relationship between ‘proactive gaze’ and MNS activity in monkeys, but their operationalisation of PG was far less restrictive than that used here (Maranesi et al., 2013). PG for their study required only target fixation prior to hand-target contact, whereas PG here also required a prior fixation on the hand/object moving toward the target. Supporters of the PG-MNS link generally invoke the direct matching hypothesis, which holds that by simulating observed actions in our mirror systems, we gain some level of direct understanding of those actions. This has also been challenged, for example by Eshuis et al. (2009), who showed that PG – in the same paradigm utilized by Falck-Ytter (2010) – was more greatly influenced by agent intention and desirability of a goal state than simply whether a human agent is moving an object. These authors argue that MNS activity may *reflect* action understanding rather than cause it, in which case searching for a strong IMR-PG relationship is unlikely to be fruitful.

Other explanations less critical of PG-MNS theory are possible. For example, if the direct matching hypothesis is accurate, our eye movements should be approximately the same whether we are observing or performing an action. For example, in one recent study regional motor activity was shown to be highly similar irrespective of whether an arm or an eye was being utilized to control a virtual object (Modroño et al., 2015). It is unlikely, however, that when performing actions we employ PG in the way operationalised herein (i.e., looking at our own hand and then predictively at the object we are reaching to grasp). Flanagan and Johansson (2003) noted in their study that gaze was rarely directed to the hand in either performance or observation of hand actions. Furthermore, when viewing videos of simple grasping actions repeatedly, ‘predictive’ gazes might soon manifest as fixations directly to the target, in which case typical PG might bypass the agent’s movement toward the object. Approximately 50% of participants here had previous exposure to the videos during the TMS component, and may have ‘known’ the outcome without the need for prediction. A final possible contributing factor concerns movement availability. PG behavior has been reported to be impaired when participants are positioned so that the execution of an observed action is not possible (Ambrosini et al., 2012). Whilst participants here did not have tied hands, they were required to be still during both TMS and eyetracking components of the study, and may not have felt in a position to perform the viewed actions, or to produce subtle head movements that might normally occur within the motor programs associated with the action.

Overall gaze preferences align well with expectations based on knowledge regarding MNS network structure and function. Increased fixation counts in hand movement AOIs tended to be positively related to IMR, in contrast to the negative associations observed with object AOI fixation counts. The strongest distinction in this regard was between Mug Body (negatively related) and Total Hand and Spoon Handle (both positively related) in the Tea Stir video. In the present context, the Spoon Handle AOI is largely a site of biological motion, as the hand grasps the handle to stir for much of the video. The key structure that feeds visual input into MNS areas – the STS – is thought to be involved in the integration and ‘interpretation’ of biological motion cues (Saitovitch et al., 2012). Reduced biological motion perception has been associated with hypoactivity in the STS and parietal mirror regions (Freitag et al., 2008). In individuals who display a relative lack of preference for biological over non-biological visual stimuli, this may be associated with aberrant STS and subsequent MNS functioning.

A less expected finding was the direction of order effects in terms of IMR. It might be predicted that participants completing the eyetracking tasks first would exhibit reduced IMR during the subsequent TMS component, due to habituation or fatigue effects after repeated earlier viewings of the images. The reverse pattern was found. Participants completing eyetracking tasks first displayed significantly greater IMR profiles on average, with facilitation generally greater during the early pulse versions of the videos. This is also unexpected as these are typically associated with reduced MEP facilitation compared to the late-pulse versions (Enticott et al., 2012a). Thus, our results may suggest a ‘priming’ or ‘learning’ effect of prior viewings. In terms of MNS theory, this does not imply a straightforward Hebbian or associative learning effect of the type advocated by Heyes (2010), which would require repeated sensory-motor pairings to instantiate a conditioned facilitation effect. Rather, it might support a model that emphasizes either top–down (Csibra, 2008) or bi-directional relationships with higher cognitive processes (Kilner et al., 2007). According to Csibra (2008), MNS function depends on prior intention ascription and conceptual processing. This process might be enhanced in the present context of repeated viewings, so that the ‘predictive validity’ of each intention ascription is gradually enhanced, leading to augmented MNS activity. Alternatively, repetition of the same grasping stimuli may lead to a short term enhancement in excitability – a ‘priming’ of the relevant neural networks. For example, Kilner et al. (2004) found that the knowledge of an upcoming observed hand movement enhanced the excitation of the observer’s own motor system, increasing their ‘readiness potential.’ The present videos were certainly more predictable for Group 2 (who did eyetracking then TMS) when their MEPs were measured, which may partially explain their strong/early response patterns.

There were several limitations to this study, the most notable being the separation of TMS and eyetracking components. Ideally, participants would undergo eyetracking analysis and TMS simultaneously. However, the Eyelink II headstrap prevented TMS coil access to the primary motor cortex, precluding in-time gaze pattern and IMR comparison and thereby reducing correlational accuracy. Whilst this issue was partially addressed through a counterbalanced design and a separate test–retest group for the eyetracking component, there is cause for caution. In addition to the order effects for IMR, there were weak test–retest correlations in some eyetracking variables. Separate Group 1 (TMS first) and Group 2 (eyetracking first) IMR-AOI correlations yielded no significant relationships at the corrected alpha level. Although this partly reflects reduced power, inferences based on overall IMR-AOI correlations here need to be carefully considered.

Second, the assumption that MEP facilitation reflects mirror neuron activity has been challenged (Dinstein et al., 2008; Hickok, 2009). The premotor cortex is understood to contain more canonical neurons than mirror neurons, and these are object-responsive (Nelissen et al., 2005). If canonical neurons activate in response to the mere presence of objects, this raises the question of whether the static hand alone is an adequate control, or whether both an object and static hand would be the minimum baseline stimuli required to isolate ‘pure’ mirror neuron activity. Whilst there was no difference between MEP responses to ‘static hand only’ and ‘static hand plus mug’ stimuli in one study (Enticott et al., 2010), there was a weak, non-significantly greater MEP response in the latter condition. The exact contribution of canonical neurons to the premotor ‘mirror response’ remains unclear. Furthermore, only the left cerebral hemisphere was examined, which is potentially significant given that left and right ventral premotor cortices may have divergent functional roles (Kilner, 2009).

There were also several eyetracking limitations. An exploratory analysis of AOI fixation *percentages* suggested a similar trend to that observed with the fixation *count* data, yet only one relationship (Mean MEP and Spoon Handle fixation percent) remained significant at the adjusted alpha level. It is possible that fixation counts reflect general attentional factors or saccadic activity rate rather than (or as well as) interest in particular AOIs. Whilst the reduced relationship strengths in fixation percentages are a concern, they are reassuring to the extent that they confirm the directionality of fixation count relationships. An alternative approach would be to measure dwell times (percent and total) in each AOI. However, ‘vacant’ or ‘blank’ staring can produce misleading dwell profiles (particularly in the context of numerous repetitive images), and fixation numbers were felt to better reflect engaged visual attention in AOIs. Finally, the AOIs were created with hand-movement versus object distinctions in mind. However, in the static/grasping videos in particular, hands move through Total Hand AOIs relatively quickly, and interact with the objects for longer periods, so that there is hand overlap in some object AOIs, potentially blurring the intended hand/object distinction. The rapid movement through hand AOIs precipitated minimal PG in the static/grasping hand videos.

In terms of more general limitations, a failure to meet parametric assumptions meant that non-parametric correlational analyses were utilized rather than regression models. Nor did this study control for IQ or concentration levels during testing. These are all shortcomings worth redressing in future studies, which would also benefit from measuring MEPs and eyetracking variables simultaneously (by using headstrap-free eyetracking equipment while applying TMS), utilizing a large sample with ASD and appropriate controls (including other developmental and/or language delay groups), and employing stimuli of a broad enough visual scope to allow greater scanning opportunity during observation of the transitive action. To the author’s knowledge, the present study is the first to explore and find a link between gaze patterns and inferred MNS activity in humans. The findings provide some (albeit qualified) support for the possibility that MNS atypicalities may be influenced by visual processes such as relative preferences for objects and biological motion.

## Conflict of Interest Statement

The authors declare that the research was conducted in the absence of any commercial or financial relationships that could be construed as a potential conflict of interest.
