# Corrigendum: Exploring associations between gaze patterns and putative human mirror neuron system activity

**DOI:** 10.3389/fnhum.2015.00523

**Published:** 2015-09-23

**Authors:** Peter H. Donaldson, Caroline Gurvich, Joanne Fielding, Peter G. Enticott

**Affiliations:** ^1^School of Psychological Sciences, Monash UniversityClayton, VIC, Australia; ^2^Monash Alfred Psychiatry Research Centre, The Alfred and Central Clinical School, Monash UniversityMelbourne, VIC, Australia; ^3^Cognitive Neuroscience Unit, School of Psychology, Deakin UniversityBurwood, VIC, Australia

**Keywords:** transcranial magnetic stimulation, mirror neurons, motor resonance, autism, gaze pattern, predictive gaze

Figure 1 of this paper contains an error in which the details for Group 1 were repeated, and the details for Group 2 were not included. This is rectified in Figure 1 included with this corrigendum.

**Figure 1:**
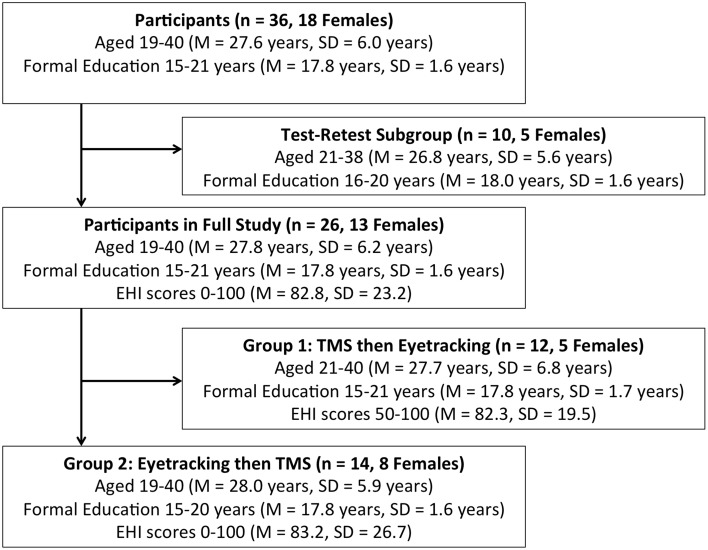
**Participant demographics and subgroup assignment**. EHI, Edinburgh Handedness Inventory; TMS, transcranial magnetic stimulation.

The original article was updated.

## Conflict of interest statement

The authors declare that the research was conducted in the absence of any commercial or financial relationships that could be construed as a potential conflict of interest.

